# Professional beliefs of physicians and allied health professionals and their willingness to promote health in primary care: a cross-sectional survey

**DOI:** 10.1186/s12875-024-02412-6

**Published:** 2024-05-27

**Authors:** Sophie Karoline Brandt, Stefan Essig, Andreas Balthasar

**Affiliations:** 1 Faculty of Health Sciences and Medicine, University of Lucerne, Lucerne, Switzerland; 2 Center for Primary and Community Care, Faculty of Health Sciences and Medicine, University of Lucerne, Lucerne, Switzerland; 3 Interface Policy Studies Research Consulting, Lucerne, Switzerland

**Keywords:** Professional beliefs, Salutogenetic, Health promotion, Willingness, Physicians, Allied health professionals, Primary care

## Abstract

**Background:**

Primary care professionals could play a key role in health promotion implementation. A fundamental aspect that might affect the willingness of primary care professionals to strengthen health promotion, and about which we do not yet know much, are professional beliefs. Therefore, we conducted a quantitative survey to (1) compare professional beliefs and the willingness to work more in health promotion between five major primary care professions, and (2) investigate associations between professional beliefs and the willingness to work more in health promotion.

**Methods:**

A large-scale cross-sectional study based on a nation-wide web-based survey of primary care professionals in Switzerland was conducted from January to July 2022. The survey was addressed to pharmacists, physicians, medical practice assistants, nurses, and physiotherapists working in primary care in Switzerland. Differences between groups were tested using T-tests and Chi-square tests. Multivariable logistic regression analyses were used to evaluate the association between variables related to professional beliefs and the willingness to work more in health promotion.

**Results:**

The responses of 4’063 primary care professionals were used for analysis. Most primary care professionals revealed a salutogenetic attitude towards their primary care tasks. Members of all professions showed high awareness of their tasks in tackling increased risks of disease (80.2% of all participants). Especially allied health professionals wished to see a greater role of prevention in primary care (pharmacists: 72.4%, medical practice assistants: 63.9%, nurses: 75.6%, physiotherapists: 73.9% versus physicians: 46.9%). All professional groups showed a high willingness to work more in health promotion (88% of all participants). Salutogenetic beliefs of primary care professionals and their willingness to work more in health promotion are strongly associated. Participants agreeing that health promotion should play a greater role or that preventive consultations should be offered in primary care, are more willing to work more in health promotion compared to participants who disagree with these ideas.

**Conclusions:**

Both affiliation to allied primary care professions and salutogenetic professional beliefs are associated with higher willingness to work more in health promotion. The high willingness provides evidence of a large, yet untapped potential. Promoting salutogenetic beliefs might further increase the willingness to engage in health promotion.

**Supplementary Information:**

The online version contains supplementary material available at 10.1186/s12875-024-02412-6.

## Background

Health promotion is defined in the WHO Ottawa Charter as the process of enabling people to gain more control over their health and to improve their health [1]. A more nuanced definition describes health promotion as an activity that mobilises healthy or protective factors to contribute to both increased resistance to disease and faster recovery from disease [2]. In the literature, health promotion is described using a positive conception of health [3] with a holistic view that takes an individual with its environment and the whole population into account [4–6]. Health promotion interventions can reduce the risk and incidence of non-communicable diseases [7–10]. Examples include behavioural counselling for nutrition and physical activity, and preventive medication [11, 12]. Although health promotion and disease prevention can be distinguished conceptually, in practice the measures of both concepts differ little from each other [6]. This is because most measures, for example in the context of primary care, ultimately pursue both the purposes of health promotion and disease prevention. For our research in this context, the distinction is therefore of little relevance.

When applying and providing health promotion activities, primary care professionals play a key role [13]. Primary care professionals are accessible for the population and maintain a trustful relationship with their patients [2, 13–15]. They provide continuity of care and can thus also provide support for lifestyle adjustments [15]. Nevertheless, health promotion is not yet sufficiently implemented in primary care [16]. There is a significant imbalance between treating disease and promoting health in healthcare provision [17, 18]. Underlying reasons were found in primary care professionals’ daily work and include lack of time, insufficient reimbursement and heavy workload [2, 14, 19, 20].

A fundamental aspect that might affect the participation of primary care professionals in health promotion activities, and about which we do not yet know much, are professional beliefs. Professional beliefs describe what professionals understand as their underlying professional tasks. These beliefs shape the approach, values and behaviour of primary care professionals and determine their interactions with patients, colleagues, and the health system as a whole [16]. Following the findings of a systematic review on barriers and facilitators to health promotion [16], professional beliefs are considered in relation to viewing risk as a disease or not, the effectiveness or efficiency of health promotion interventions, adverse effects of risk assessment and the medicalisation of life, the use of medication for prevention, which patients might benefit and who should be responsible for health promotion interventions. Professional beliefs can be divided into two opposing views: The pathogenetic perspective and the salutogenetic perspective. According to the pathogenetic perspective, primary care professionals primarily alleviate or cure diseases [21]. The perspective also includes that increased risk for disease does not need to be treated [16, 21]. According to the salutogenetic perspective, primary care professionals should maintain and promote the health of patients [21]. This includes a holistic perspective of primary care professionals on patients and factors that promote their health [21]. These beliefs influence both motivation and attitudes towards health promotion [16].

Previous research on professional beliefs and primary care professionals’ health promotion activities in primary care is limited. So far, studies have mainly been conducted with qualitative methods [12, 19, 20, 22–24]. They found that general practitioners’ own health habits and attitudes towards health promotion could have an impact on the implementation of health-promoting care [20]. The few quantitative studies hypothesise that the involvement of allied health professionals in primary care practices contributes to more health promotion activities in primary care [25]. There has been no quantitative research into whether professional beliefs affect the willingness of primary care professionals to work more in health promotion. Another limitation is that previous studies have focused on general practitioners and nurses [14, 19, 20, 22, 26–28], as only two groups of primary care professionals. Little is known about the professional beliefs of pharmacists, medical practice assistants, and physiotherapists as large and increasingly important professional groups in primary care. Compared to previous qualitative studies, our quantitative, large-scale study offers the added value of providing conclusive findings on professional beliefs of many primary care professionals from different professional groups and with different educational and regional backgrounds. It also bridges the knowledge gap on whether professional beliefs influence the willingness of primary care professionals to work more in health promotion and thus to fulfil their key role in health promotion activities.

Therefore, we conducted a quantitative survey to (1) compare professional beliefs and the willingness to work more in health promotion between five major primary care professions, and (2) investigate associations between professional beliefs and the willingness to work more in health promotion.

## Methods

### Study design, setting and participants

A large-scale cross-sectional study based on a nation-wide web-based survey of primary care professionals in Switzerland was conducted [29, 30]. Data were collected over a period of 7 months (January - July 2022).

The survey was addressed to pharmacists, physicians, medical practice assistants, nurses, and physiotherapists working in primary care. They are the five largest professional groups of primary care professionals in Switzerland. In Swiss primary care, the professional group of medical practice assistants is responsible for administrative tasks and selected medical-technical activities related to diagnostics and therapy in general practitioner (GP) practices [31]. After completing further training, they can also take on additional tasks under medical supervision, such as advising patients with chronic diseases [32, 33].

A non-probabilistic sampling procedure was used due to challenges in the access to the primary care professionals. Access to professionals followed the recommendations by Alvarez and VanBeselaere [34] and was achieved through different partners and media channels. The partners include professional and specialist associations, pharmacy, and practice networks, interprofessional organisations as well as training and research institutions. These informed their members about the survey via newsletter, intranet, member magazine or member mail and distributed the link to the survey. After about four to eight weeks, professional associations and networks sent out a reminder by email or published an article on the survey in their member magazine to encourage as many professionals as possible to participate. There was no financial incentive for professionals to fill in the survey. As the survey was also distributed via media that are accessible to everyone, it was not possible to prevent people without a medical or healthcare profession who were interested in the topic from taking part. These could have been e.g., patients, managers, or administrative staff.

### Measurement

The survey used was a structured questionnaire developed for the study and consisting of close-ended questions (Supplementary Table 1, Additional File 1). Pre-tests were conducted with survey experts and professionals from the professional groups addressed using the think-aloud protocol method [35]. Questions that lacked clarity or comprehensiveness were revised accordingly. All information was self-reported. The following sociodemographic characteristics were surveyed: Sex, age, profession, professional experience, type of employment, and region of work. Age and professional experience were defined as continuous variables. The primary care professionals from the five selected professional groups were identified using filter questions about their profession.

The type of employment was surveyed in four categories (employed with management responsibility, employed without management responsibility, self-employed with employees, self-employed without employees). The region of work was surveyed in three categories (urban, intermediate, rural).

The question regarding willingness to work more health-promoting (“How willing are you to do more health-promoting and preventive work in your current position?”) was closely aligned with the question that has already been used by Johansson et al. [2]. The response options were on a 5-point Likert scale (very low, low, neither low nor high, high, very high). Following findings from Tengland [6] and the measurements of Johansson et al. [2], we asked about both areas health promotion and disease prevention as their measures can rarely be distinguished in practice.

The professional beliefs were measured using three statements derived from investigations by Johansson et al. [2], Pelikan [21] and Rubio-Valera et al. [16]. Johansson et al. [2] have examined the extent to which primary care professionals were generally committed to a more health-promoting health service. This led to the question “To what extent do you agree with the following statement: Prevention should not play a greater role in primary care than the treatment of diseases.”. Pelikan [21] and Rubio-Valera et al. [16] have found that the professionals’ attitudes that treating increased risk of disease is not a professional responsibility could serve as a barrier for professionals in implementing health promotion activities. This finding led to the two questions “To what extent do you agree with the following statement: The task of primary care professionals includes treating diseases. Treating increased risks for diseases is not part of their tasks.” and “Your neighbour thinks that primary care should only be used to treat diseases. He does not think that preventive examinations and preventive consultations should be offered in primary care for people with increased risks of disease. To what extent do you agree with him?”. The participants had to rate their answer on a 5-point Likert scale (strongly disagree, disagree, neutral, agree, strongly agree). With regard to the interpretation of the responses, we followed the results of Johansson et al. [2], Pelikan [21] and Rubio-Valera et al. [16]. For this purpose, professionals who agreed or strongly agreed with the respective statement were considered pathogenetically orientated. Professionals who disagreed or strongly disagreed with the respective statement were considered to have a salutogenetic perspective.

Participants took approximately 17 min to complete the questionnaire. This duration arose because other questions were asked on other topics that are not further addressed in this publication. The survey was programmed using Qualtrics software, Version January 2022 (Qualtrics, Provo, UT, USA).

Informed consent was obtained from all participants. Consent was obtained by informing participants on the first page, prior to completing the questionnaire, about the research purpose of the study, the voluntariness of participation with the option to withdraw at any time, and the confidentiality of the information they provided.

### Statistical methods

The sociodemographic characteristics were regarded as independent variables. Professional groups were defined as categorical variable (pharmacists, physicians, medical practice assistants, nurses, and physiotherapists. The three variables on professional beliefs were also regarded as independent variables and split from originally five into three levels (agree, neutral, disagree) to increase the number of responses per level. As relatively few participants agreed or strongly agreed that the treatment of risks is not the task of professionals or that preventive examinations and preventive consultations should not be offered in primary care, this recoding prevented random error in the analysis. The responses “strongly disagree” and “disagree” were summarised to “disagree”. The responses “strongly agree” and “agree” were summarised to “agree”. The variable on willingness to work more in health promotion was considered as dependent variable and split into three levels (low, neither low nor high, high). The responses “very low” and “low” were summarised to “low”. The responses “very high” and “high” were summarised to “high”.

Discrete variables were presented with tables showing the number and percentage distributions. The continuous variable was described using mean and standard deviation (SD). The five professional groups within the study population were compared using cross tables. T-test and Chi-square test were used to test for differences between groups.

Ordered logistic regression analyses were used to evaluate associations between professional beliefs and the willingness to work more in health promotion. The confidence interval was set at 95% and *p*-value less than 0.05 was considered statistically significant. The respective reference category for the calculation of odds ratios (OR) is mentioned in the presentation of the ordered logistic regression results. Since there were only a few missing values, no imputation had to be made. After crude analysis, we then adjusted the regression model for sociodemographic characteristics including age and sex (basic model) and additionally profession, professional experience, type of employment, and region of work, (extended model). We conducted sensitivity analyses to assess the robustness of our ordered logistic regression results, reclassifying “neutral” responses as “disagree” for the variables related to professional beliefs. Data analyses were performed using R Statistical Software, Version 4.2.2 [36].

## Results

### Participants

A total of 5’014 participants took part in the survey. After excluding participants who did not work in a medical or health profession (118), who did not provide information about their professional background (385) or who belonged to professional groups that were not included in the study (448), the adjusted sample is based on 4’063 participants.

Of all participants, 17.7% were pharmacists, 14.9% were physicians, 19.8% were medical practice assistants, 15.8% were nurses and 31.8% were physiotherapists. Around 10% of the pharmacists, 3% of the physicians in primary care, 4% of medical practice assistants, 3% of the nurses and 10% of the physiotherapists practising in Switzerland took part in the survey [37–40]. Although we could not expect a sample representative of the target population due to the way participants were contacted, the sex and age distribution within the sample across all professional groups largely corresponds to the target populations [39, 41].

Regarding sociodemographic characteristics of the study population, all tests for differences between the groups were statistically significant (*p* < .01) (Table 1). Most of the participating pharmacists, physicians, nurses, and physiotherapists were over 46 years of age and older. Among the medical practice assistants, most were under 30. The physicians surveyed had the most professional experience and the medical practice assistants the least. On average, slightly less than half of the participants worked in urban regions, while the other half worked in intermediate or rural regions.

**Table 1:** Sociodemographic characteristics of study population

	Total	Pharmacists	Physicians	Medical practice assistants	Nurses	Physiotherapists	*P*
**Total**	4063	720	606	803	643	1291	
**Sex (%)**							< 0.001
Female	3187 (78.5)	535 (74.3)	274 (45.3)	796 (99.1)	570 (88.6)	1012 (78.4)	
Male	862 (21.2)	180 (25.0)	328 (54.2)	6 (0.7)	73 (11.4)	275 (21.3)	
Diverse	12 (0.3)	5 (0.7)	3 (0.5)	1 (0.1)	0 (0.0)	3 (0.2)	
**Age groups (%)** ^a^							< 0.001
30 and younger	794 (19.6)	74 (10.3)	14 (2.3)	394 (49.2)	106 (16.5)	206 (16.0)	
31–45	1369 (33.8)	273 (37.9)	144 (23.8)	261 (32.6)	180 (28.0)	511 (39.7)	
46 and older	1892 (46.7)	373 (51.8)	446 (73.8)	146 (18.2)	357 (55.5)	570 (44.3)	
**Professional experience in years on average (SD)**							< 0.001
Overall	14.82 (6.40)	14.7 (6.4)	17.7 (4.3)	11.8 (7.1)	16.3 (5.6)	14.2 (6.5)	
In primary care	11.20 (7.52)	11.3 (7.8)	14.1 (6.3)	11.1 (7.2)	6.7 (7.3)	11.9 (7.2)	
**Type of employment (%)**							< 0.001
Employed with management responsibility	1098 (36.3)	337 (60.4)	96 (19.9)	234 (48.9)	224 (46.5)	207 (20.2)	
Employed without management responsibility	1049 (34.6)	126 (22.6)	55 (11.4)	233 (48.6)	210 (43.6)	425 (41.4)	
Self-employed with employees	548 (18.1)	88 (15.8)	307 (63.7)	11 (2.3)	14 (2.9)	128 (12.5)	
Self-employed without employees	333 (11.0)	7 (1.3)	24 (5.0)	1 (0.2)	34 (7.1)	267 (26.0)	
**Region of work (%)**							< 0.001
Urban	1407 (46.4)	269 (48.1)	212 (43.7)	200 (41.7)	210 (43.6)	516 (50.2)	
Intermediate	805 (26.5)	164 (29.3)	153 (31.5)	92 (19.2)	129 (26.8)	267 (26.0)	
Rural	822 (27.1)	126 (22.5)	120 (24.7)	188 (39.2)	143 (29.7)	245 (23.8)	

^a^ To illustrate age differences of the participants between professional groups, age is presented by category

Percentages were calculated from the number of respondents to each question

SD: Standard deviation

### Professional beliefs and willingness to work more health-promoting

Physicians showed a particularly high awareness of their tasks in tackling increased risks of disease (Table 2). Only a few of them stated that treatment of increased risks of disease is not one of the tasks of primary care professionals (3.9%). This low proportion of opposing views was shared by most allied health professionals: 6.5% of nurses, 11.3% of physiotherapists and 13.1% of pharmacists. Among medical practice assistants, 20% shared this view.

While allied health professionals wished to see a greater role of prevention in primary care, the physicians’ view was less clear. Slightly less than half (46.9%) supported a greater role for prevention, while the other half were neutral or positive.

Most participants indicated that preventive consultations should be offered in primary care for people at increased risk of disease. In the group of pharmacists, physicians, nurses, and physiotherapists, only 2% or less indicated that no preventive services should be offered. Of the medical practice assistants, 10.6% indicated the same or were neutral.

The respondents showed a high to very high willingness to work more in health promotion (Table 2). All tests for differences between the groups are statistically significant (*p* < .001). The willingness is high among all participants: pharmacists (90.4%), physicians (80.5%), medical practice assistants (83.5%), nurses (90.9%), and physiotherapists (90.9%). More physicians showed neither low nor high willingness (16.6%) compared to allied health professionals.

**Table 2:** Professional beliefs and willingness to work more in health promotion

	Total		Pharmacists	Physicians	Medical practice assistants	Nurses	Physiotherapists	*P*
“Prevention should not play a greater role in primary care than the treatment of diseases.” (%)								< 0.001
Disagree	2101 (68.0)		411 (72.4)	229 (46.9)	319 (63.9)	371 (75.6)	771 (73.9)	
Neutral	606 (19.6)		101 (17.8)	145 (29.7)	117 (23.4)	77 (15.7)	166 (15.9)	
Agree	383 (12.4)		56 (9.9)	114 (23.4)	63 (12.6)	43 (8.8)	107 (10.2)	
“The task of primary care professionals includes treating diseases. Treating increased risks for diseases is not part of their tasks.” (%)								< 0.001
Disagree	2474 (80.2)		434 (76.7)	454 (92.7)	314 (62.9)	430 (87.9)	842 (80.9)	
Neutral	268 (8.7)		58 (10.2)	17 (3.5)	85 (17.0)	27 (5.5)	81 (7.8)	
Agree	343 (11.1)		74 (13.1)	19 (3.9)	100 (20.0)	32 (6.5)	118 (11.3)	
“Your neighbour thinks that primary care should only be used to treat diseases. He does not think that preventive examinations and preventive consultations should be offered in primary care for people with increased risks of disease. To what extent do you agree with him?” (%)								0.002
Disagree	2888 (93.6)		543 (95.8)	459 (94.4)	444 (89.3)	466 (95.1)	976 (93.4)	
Neutral	140 (4.5)		15 (2.6)	21 (4.3)	36 (7.2)	20 (4.1)	48 (4.6)	
Agree	57 (1.8)		9 (1.6)	6 (1.2)	17 (3.4)	4 (0.8)	21 (2.0)	
Willingness to work more in health promotion and prevention (%)								< 0.001
Low		72 (2.3)	8 (1.4)	14 (2.9)	15 (3.0)	13 (2.7)	22 (2.1)	
Neither low nor high		299 (9.7)	46 (8.2)	81 (16.6)	67 (13.5)	32 (6.5)	73 (7.0)	
High		2708 (88.0)	510 (90.4)	393 (80.5)	414 (83.5)	444 (90.8)	947 (90.9)	

Percentages were calculated from the number of respondents to each question

### Associations between professional beliefs and willingness to work more in health promotion

In the three models of the regression analyses, between 2972 and 3044 observations were included (Table 3). The predictors of the extended model explain 20.7% of variance in the outcome variable. The regression results indicate that there are statistically significant relationships between most independent variables and the professionals’ willingness to work more in health promotion.

Looking at the professional beliefs, participants with a neutral view on whether prevention should play a greater role in primary care than the treatment of diseases, had 0.58 times the odds of high willingness to work more in health promotion (95% CI 0.43–0.78, *p* < .001) compared to participants who disagreed that health promotion should not play a greater role in primary care. The negative association was even stronger for participants who agreed with this idea; they have 0.28 times the odds of high willingness to work more health-promoting (95% CI 0.21–0.38, *p* < .001) compared to participants who disagree.

Those who had a neutral view on whether increased risks should be treated by primary care professionals had 43% lower odds of high willingness to work more in health promotion (95% CI 0.39–0.83, *p* < .01) compared to participants who disagreed that increased risks should not be treated by primary care professionals, professional beliefs held constant.

Participants with a neutral view on whether preventive consultations should be offered in primary care for people at increased risk of disease had 0.17 times the odds of high willingness to work more in health promotion (95% CI 0.12–0.25, *p* < .001) compared to participants who disagreed that preventive services should be offered. Furthermore, participants agreeing that preventive consultations should not be offered in primary care have 77% lower odds of high willingness to work more in health promotion (95% CI 0.13–0.42, *p* < .001) compared to participants who disagree that preventive consultations should not be offered in primary care.

The sensitivity analyses showed the robustness of our results. The odds ratios and significance levels remained largely consistent despite the change in categorisation of the variables on professional beliefs (Supplementary Table 2, Additional file 2).

**Table 3:** Odds ratios and confidence intervals for high willingness to work more in health promotion by professional beliefs

	High willingness to work more in health promotion					
	Crude analysis		Basic model^a^		Extended model^b^	
Predictors	OR (95% CI)	*P*-value	OR (95% CI)	*P*-value	OR (95% CI)	*P*-value
**Professional beliefs**						
“Prevention should not play a greater role in primary care than the treatment of diseases.”: Reference = Disagree						
Neutral	0.51 (0.39–0.68)	< 0.001	0.52 (0.39–0.69)	< 0.001	0.58 (0.43–0.78)	< 0.001
Agree	0.23 (0.17–0.30)	< 0.001	0.24 (0.18–0.31)	< 0.001	0.28 (0.21–0.38)	< 0.001
“The task of primary care professionals includes treating diseases. Treating increased risks for diseases is not part of their tasks.”: Reference = Disagree						
Neutral	0.57 (0.41–0.82)	0.002	0.54 (0.39–0.78)	0.001	0.57 (0.39–0.83)	0.003
Agree	0.78 (0.55–1.12)	0.167	0.77 (0.54–1.12)	0.159	0.77 (0.53–1.12)	0.164
“Your neighbour thinks that primary care should only be used to treat diseases. He does not think that preventive examinations and preventive consultations should be offered in primary care for people with increased risks of disease. To what extent do you agree with him?”: Reference = Disagree						
Neutral	0.16 (0.11–0.23)	< 0.001	0.16 (0.11–0.24)	< 0.001	0.17 (0.12–0.25)	< 0.001
Agree	0.23 (0.13–0.42)	< 0.001	0.24 (0.14–0.44)	< 0.001	0.23 (0.13–0.42)	< 0.001
Observations	3044^c^		3033^c^		2972^c^	
R^2^ Nagelkerke	0.157		0.166		0.207	

^a^ adjusted for age and sex

^b^ adjusted for age, sex, profession, professional experience, type of employment, and region of work

^c^ The difference from the total number of participants is due to missing answers to questions regarding sociodemographic characteristics or professional beliefs

OR: Odds ratio, CI: Confidence interval

## Discussion

In this study, we compared professional beliefs and the willingness to work more in health promotion between five major primary care professions and investigated associations between professional beliefs and the willingness to work more in health promotion.

Primary care professionals showed that they have a salutogenetic attitude towards their primary care tasks and do not strongly support the biomedical pathogenetic model. Physicians and allied health professionals showed a high awareness of their tasks in tackling increased risks of disease. However, while allied health professionals requested a stronger role of prevention in primary care, physicians were less supportive of this stronger role of prevention. Most participants indicated that preventive consultations should be offered in primary care for people at increased risk of disease. Most opponents of the treatment of increased risks for diseases and the greater role of health promotion in primary care were found among medical practice assistants compared to all other professional groups. All professional groups showed a high willingness to work more in health promotion. This willingness was particularly high among pharmacists, nurses, and physiotherapists and the lowest among physicians and medical practice assistants.

Salutogenetic beliefs of primary care professionals and their willingness to work more in health promotion are strongly associated. This mainly concerns the professional beliefs according to which prevention should not play a greater role in primary care than the treatment of diseases and preventive examinations and preventive consultations should not be offered in primary care for people with increased risks of disease. Participants who agreed that health promotion should play a greater role in primary care, have higher odds of high willingness to work more in health promotion compared to participants who disagree with this idea. Participants agreeing that preventive consultations should be offered in primary care have higher odds of high willingness to work more in health promotion compared to participants who disagree that preventive consultations should be offered in primary care. The association between the professional belief according to which treating increased risks for diseases is not part of primary care professionals’ tasks and willingness to work more in health promotion is less strong.

Our findings for physicians showing a lower willingness to implement health promotion activities compared to allied health professionals are consistent with Johansson et al. [2]. In their study that surveyed health professionals in a Swedish province, they similarly found that physicians showed less commitment to more health promotion in health services in general compared to other primary care professionals. Our analysis does not allow us to conclude that these differences between the professional groups are due to pathogenetic-oriented professional beliefs of physicians. Hypothetically, a more pathogenetic-oriented training and remuneration of physicians might have an effect [16, 21].

The assumption regarding pathogenetic-oriented training is also raised in other studies that report suboptimal and unsystematic implementation of health promotion or preventive care by physicians [20] or a lack of skills to offer health promotion [19, 27]. In contrast, allied health professionals such as dieticians, occupational therapists, physiotherapists, and psychologist reported to feel competent to promote their patients’ health [2].

Bock et al. [27] provide evidence that physicians become more committed to health promotion with increasing age. These results might show the impact of professional experience on the health professionals’ commitment to deliver prevention and health-promoting services. Most physicians in our sample are over 46 years old and have on average 17.7 years of professional experience, so this effect might also have occurred in our study.

All professional groups show a high degree of willingness to work more in health promotion. Our findings for allied primary care professionals indicating a specifically high willingness are also in line with previous studies. Johansson et al. [2] found that psychologists, occupational therapists, and physiotherapists reported their health-promoting commitment most frequently compared to nurses and physicians. The high willingness of primary care professionals to strengthen health promotion, therefore, has a lot of potential that remains untapped. It remains unclear why this potential is not being realised. Johansson et al. [2] and Rubio-Valera et al. [16] provide evidence that daily work factors, such as work overload or missing patients’ interests, could be potential starting points. Regarding the allied primary care professionals potential, evidence suggests that the current healthcare system does not ask for their competences in health promotion [2].

In terms of professional beliefs, our findings for physicians showing a high awareness of their roles in tackling increased risks of disease are consistent with the findings of Schneider et al. [28]. In their survey of German physicians, more than 95% of respondents agreed that it is their role not only to treat disease but also to act as health advisors. However, the authors argue that physicians in Germany spend the least consultation time per patient compared to physicians in Switzerland and four other European countries [42]. This may not be enough time to implement health promotion effectively [28]. As Germany and Switzerland are neighbouring countries and face similar challenges in terms of demographic change, sharing physicians’ awareness of their responsibilities in tackling increased risks of disease is an important prerequisite for health promotion.

Previous research has shown that understanding of professional beliefs varies depending on the focus of the studies. In studies focusing on specific study populations such as patients with type 2 diabetes [43] or with lymphoedema [44], professional beliefs were often interpreted as health professionals’ experiences. In studies focusing on specific tasks or activities of health professionals, e.g. domestic violence disclosure [45], professional beliefs were considered as attitudes of health professionals. In these studies, health professionals’ salutogenetic and pathogenetic perspectives were not discussed as factors influencing professional beliefs.

Previous studies on health promotion and the role of professional beliefs have examined the treatment of certain diseases or unhealthy habits such as smoking. For example, Tong et al. [46] found that health professionals who believed that treatment was an important professional task were more likely to provide tobacco use counselling. Conversely, they also found negative associations of tobacco use counselling and the belief that counselling was not an appropriate service. Although our study did not focus on specific diseases or unhealthy habits, our findings on the associations of professional beliefs and primary care professionals’ commitment to health promotion are consistent with those of Tong et al. [46].

### Implications for policy and practice

Our findings reinforce the key role of primary care professionals in the promotion of health. With this study, we provide evidence of the high willingness of primary care professionals to strengthen health promotion, supported by their salutogenetic professional beliefs. This high willingness to strengthen health promotion enables a positive understanding of health [3] and a holistic view of individuals, their environment, and the entire population [4–6] to be consolidated in primary care in the future. This offers many opportunities in primary care to mobilise healthy or protective factors that help the population to achieve increased resistance to disease and faster recovery from disease [2]. To achieve this and successfully promote the health of the population, the reasons why the potential of primary care professionals is not being used should be investigated.

On the one hand, our research findings can help those responsible for health policy strategies and health promotion projects to select professional groups that are particularly motivated to implement their programmes. On the other hand, they can support decision-makers and educators in adapting working conditions and training programmes in such a way that they positively influence professional beliefs and thus the willingness to work more in health promotion.

Our results suggest that policymakers, healthcare managers and educators should support the engagement of primary care professionals in promoting public health in several ways. First, it is important to encourage primary care professionals to adopt a salutogenetic attitude towards their primary care tasks. Efforts to do this should be reinforced, especially among physicians and medical practice assistants, for example in the context of their training and working conditions. Secondly, it should primarily be made possible for allied health professionals to take on a leading role in the field of health promotion because they have a strong desire for health promotion to play a more important role in primary care and they are willing to strengthen health promotion.

### Study strengths and limitations

This is the first study measuring the association between professional beliefs and primary care professionals’ willingness to strengthen health-promotion. Furthermore, this investigation is relevant in addressing persisting problems in health promotion activities in primary care in Switzerland. The survey of a broad population of primary care professionals with different professional backgrounds and sociodemographic characteristics was successful thanks to the support of many public and private stakeholders in Swiss primary care and led to a large study size. The quantitative study design made it possible to compare willingness to strengthen health-promotion and professional beliefs between different professional groups.

Intensive efforts were undertaken to disseminate our survey and to encourage as many primary care professionals as possible to participate in our study. This was done with the aim of implementing the principle of probability sampling. In terms of sex and age distribution, our sample corresponds to the target population. Nevertheless, we cannot completely exclude the possibility of selection bias in our data [47]. Since our study design involves the analysis of self-reports, we can equally not exclude random measurement error and self-report bias [48]. To minimise both in the measurement of professional beliefs, we used three different questions with different thematic focuses.

We took various measures to minimise the impact of potential social desirability bias on the results of our study. Following the recommendations of Converse et al. [49] and Peabody et al. [50], we wanted to ensure that the respondents did not interpret any particular answer as the only desired answer. For this purpose, we assessed professional beliefs using three different questions with different emphases. We asked about willingness to work more in health promotion in the current professional position rather than willingness in general. In this way, we intended to direct the respondents’ focus on their current position and not on their ideal, socially desirable professional position in which they could exercise best practice. Nevertheless, we cannot exclude a possible overestimation of the real willingness to work more in health promotion and the real salutogenetic-orientated professional beliefs [49, 51].

Like all cross-sectional studies [52], our study faces the limitation of measuring exposure and outcome simultaneously at a single point in time. We cannot and did not intend to provide evidence of a causal relationship between professional beliefs and primary care professionals’ willingness to work more in health promotion. A longitudinal study would provide deeper insights into changes in professional beliefs and actual health-promoting activities of primary care professionals during their work.

Similar to Johansson et al. [2], we do not know the extent professionals considered themselves to be doing health promotion in daily practice already. Furthermore, we did not provide a general definition of health promotion in the questionnaire. We therefore cannot exclude that the participants had a different understanding of health promotion and that this had an influence on their answers. There is evidence in the literature of different perspectives on health promotion [16], but a clear distinction between the different definitions of health promotion by professional group has not yet been identified. Last, our survey has been available in German and French language leading to few participants from the canton Ticino. Consequently, we cannot transfer our results to the Italian-speaking part of Switzerland.

## Conclusion

More efforts should be made to promote a salutogenetic attitude towards their primary care tasks among physicians and medical practice assistants as part of their training and working conditions. In addition, allied health professionals in particular should be enabled to take a leading role in health promotion, as they have a strong desire for health promotion to play a more important role in primary care and are willing to strengthen health promotion.

Both affiliation to allied primary care professions and salutogenetic professional beliefs are associated with higher willingness to work more in health promotion. The high willingness provides evidence of a large, yet untapped potential. Promoting salutogenetic beliefs might further increase the willingness to engage in health promotion.

### Electronic supplementary material

Below is the link to the electronic supplementary material.

Supplementary Material 1

Supplementary Material 2

## Data Availability

The datasets used and analysed during the current study are available from the corresponding author on reasonable request.

## Abbreviations

OR: Odds ratio
CI: Confidence interval
SD: Standard deviation
