# Supplementary material for: Professional beliefs of physicians and allied health professionals and their willingness to promote health in primary care: a cross-sectional survey
Source: BMC Prim Care. 2024 May 27;25:188. doi: 10.1186/s12875-024-02412-6 (PMC11129482; doi:10.1186/s12875-024-02412-6)
Supplement: Supplementary file 1 — Supplementary Material 1 [file 12875_2024_2412_MOESM1_ESM.pdf]

Supplementary information

Additional file 1

Supplementary Table 1: Questionnaire and variables from survey «Health 2040»

Variable	Question	Response options	Used in analysis
**Sociodemographic characteristics**			
Sex	Please indicate your sex.	○ Female<br>○ Male<br>○ Diverse	Independent variable
Age	What is your year of birth?	Dropdown menu	Independent variable
Professional background	Which of the following statements apply to you?<br>Patients always include clients.	○ I work in a medical or health profession with contact to patients.<br>○ I work in a medical or health profession without contact with patients.<br>○ I do not practise a medical or	

Variable	Question	Response options	Used in analysis
		health profession.	
Profession	To which professional group do you currently belong?	○ Pharmacists<br>○ Physicians<br>○ Medical practice assistants<br>○ Nurses<br>○ Physiotherapists<br>○ Other profession in the health sector, please specify [text field].	Independent variable
Professional experience (overall)	How many years of professional experience do you have in the health sector?	Dropdown menu	Independent variable
Professional experience (in primary care)	How many years of that time do you work fully or partially in outpatient healthcare?	Dropdown menu	
Type of employment		○ Employed with management responsibility<br>○ Employed	Independent variable

Variable	Question	Response options	Used in analysis
		without management responsibility<br>○ Self-employed with employees<br>○ Self-employed without employees	
Region of work		○ Urban<br>○ Intermediate<br>○ Rural	Independent variable
**Professional beliefs**			
Professional beliefs	To what extent do you agree with the following statement: Prevention should not play a greater role in primary care than the treatment of diseases.	○ Strongly disagree<br>○ Disagree<br>○ Neutral<br>○ Agree<br>○ Strongly agree	Independent variable
Professional beliefs	To what extent do you agree with the following statement: The task of primary care professionals includes treating diseases.	○ Strongly disagree<br>○ Disagree<br>○ Neutral<br>○ Agree<br>○ Strongly agree	Independent variable

Variable	Question	Response options	Used in analysis
	Treating increased risks for diseases is not part of their tasks.		
Professional beliefs	Your neighbour thinks that primary care should only be used to treat diseases. He does not think that preventive examinations and preventive consultations should be offered in primary care for people with increased risks of disease. To what extent do you agree with him?	○ Strongly disagree<br>○ Disagree<br>○ Neutral<br>○ Agree<br>○ Strongly agree	Independent variable
**Willingness to work more in health promotion**			
Willingness to work more in health promotion	How willing are you to do more health-promoting and preventive work in your current position?	○ Very low<br>○ Low<br>○ Neither low nor high<br>○ High<br>○ Very high	Dependent variable

5 www.health2040.ch
