# Supplementary material for: Professional beliefs of physicians and allied health professionals and their willingness to promote health in primary care: a cross-sectional survey
Source: BMC Prim Care. 2024 May 27;25:188. doi: 10.1186/s12875-024-02412-6 (PMC11129482; doi:10.1186/s12875-024-02412-6)
Supplement: Supplementary file 2 — Supplementary Material 2 [file 12875_2024_2412_MOESM2_ESM.pdf]

Supplementary information

Additional file 2

Supplementary Table 2: OR and CI for willingness to work more in health promotion by professional beliefs (in 2 categories)

	**High willingness to work more in health promotion**					
	Crude analysis		Basic model[a]		Extended model[b]	
Predictors	OR (95% CI)	*P*-value	OR (95% CI)	*P*-value	OR (95% CI)	*P*-value
**Professional beliefs**						
"Prevention should not play a greater role in primary care than the treatment of diseases.": Reference = Disagree						
Agree	0.29 (0.23 – 0.38)	<0.001	0.30 (0.23 – 0.39)	<0.001	0.34 (0.26 – 0.45)	<0.001
"The task of primary care professionals includes treating diseases. Treating increased risks for diseases is not part of their tasks.": Reference = Disagree						
Agree	0.80 (0.58 – 1.14)	0.209	0.81 (0.58 – 1.15)	0.228	0.83 (0.59 – 1.19)	0.288
"Your neighbour thinks that primary care should only be used to treat diseases. He does not think that preventive examinations and preventive consultations should be offered in primary care for people with increased risks of disease. To what extent do you agree with him?": Reference = Disagree						
Agree	0.28 (0.16 – 0.49)	<0.001	0.28 (0.16 – 0.50)	<0.001	0.28 (0.16 – 0.50)	<0.001
Observations	3044		3033		2972	
R^2 Nagelkerke	0.079		0.089		0.145	

[a] adjusted for age and sex

[b] adjusted for age, sex, profession, professional experience, type of employment, and region of work

OR: Odds ratio, CI: Confidence interval
